# Applications of Next-generation Sequencing in Systemic Autoimmune Diseases

**DOI:** 10.1016/j.gpb.2015.09.004

**Published:** 2015-10-15

**Authors:** Yiyangzi Ma, Na Shi, Mengtao Li, Fei Chen, Haitao Niu

**Affiliations:** 1 Institute of Laboratory Animal Sciences, Chinese Academy of Medical Sciences and Comparative Medicine Center, Peking Union Medical Collage, Beijing 100021, China; 2 Department of Rheumatology and Clinical Immunology, Peking Union Medical College Hospital, Chinese Academy of Medical Sciences and Peking Union Medical Collage, Beijing 100730, China; 3 CAS Key Laboratory of Genome Sciences and Information, Beijing Institute of Genomics, Chinese Academy of Sciences, Beijing 100101, China

**Keywords:** Next-generation sequencing, Intestinal microbiome, Susceptibility genes, Systemic autoimmune diseases

## Abstract

**Systemic autoimmune diseases** are a group of heterogeneous disorders caused by both genetic and environmental factors. Although numerous causal genes have been identified by genome-wide association studies (GWAS), these **susceptibility genes** are correlated to a relatively low disease risk, indicating that environmental factors also play an important role in the pathogenesis of disease. The **intestinal microbiome**, as the main symbiotic ecosystem between the host and host-associated microorganisms, has been demonstrated to regulate the development of the body’s immune system and is likely related to genetic mutations in **systemic autoimmune diseases**. **Next-generation sequencing** (NGS) technology, with high-throughput capacity and accuracy, provides a powerful tool to discover genomic mutations, abnormal transcription and **intestinal microbiome** identification for autoimmune diseases. In this review, we briefly outlined the applications of NGS in **systemic autoimmune diseases**. This review may provide a reference for future studies in the pathogenesis of **systemic autoimmune diseases**.

## Introduction

Since the inception of cyclic array-based next-generation sequencing (NGS) in 2005 [1], application of this high-throughput technology has shown exponential increase in related biomedical studies. NGS can be applied to sequence analysis on any part of the genome and the resulting transcriptome, including the whole genome, exons, and other interesting regions, and accordingly can be roughly classified as whole-genome sequencing (WGS), whole-exome sequencing (WES), RNA sequencing (RNA-seq), and DNA methylation sequencing [2–4].

Systemic lupus erythematosus (SLE), rheumatoid arthritis (RA), multiple sclerosis (MS), ankylosing spondylitis (AS), and Sjögren’s syndrome (SS) are typical systemic autoimmune diseases, which affect multiple organs and exhibit inherited susceptibility. Multiple causal genes have been identified that influence the development of autoimmune disorders by genome-wide association studies (GWAS) [5]. However, each of such genes is generally associated with only a relatively low risk of autoimmune disease occurrence, indicating that presence of the identified susceptibility genes is not a definitive pre-requisite for the disease development [6,7]. According to the “hygiene hypothesis”, environmental pressure affects genetic alleles, rendering the body’s immune system to adapt to the environmental impact, including the presence of microorganisms [8]. Therefore, the simultaneous presence of susceptibility genes and the gut microbiome is likely to coordinate synergistically to promote the systemic autoimmune disease progression.

The human intestinal contains a vast and diverse microbial ecosystem, consisting of 10^14^–10^15^ microorganisms, colonizing the human intestinal tract shortly after birth, and remaining there throughout an individual’s life. Both the quantities and the species composition of intestinal microbiota are closely related to human health [9]. Each person possesses millions of microbial genes, which are around 100-fold greater than the number of human genes [10]. This microbial gene pool comprises genes from hundreds of microbial species. The majority of these bacteria fall into four phyla: Actinobacteria, Firmicutes, Proteobacteria, and Bacteroidetes [10,11].

The value of NGS has been demonstrated in identifying susceptibility genes associated with systemic autoimmune diseases [11–13]. Its efficacy has also been demonstrated in the characterization of intestinal microbiotas [12–14]. Our goal is to provide an overview of the current applications of NGS to better understand the pathogenesis of systemic autoimmune diseases.

## NGS technology is widely applied in biomedical research

**Figure 1:**
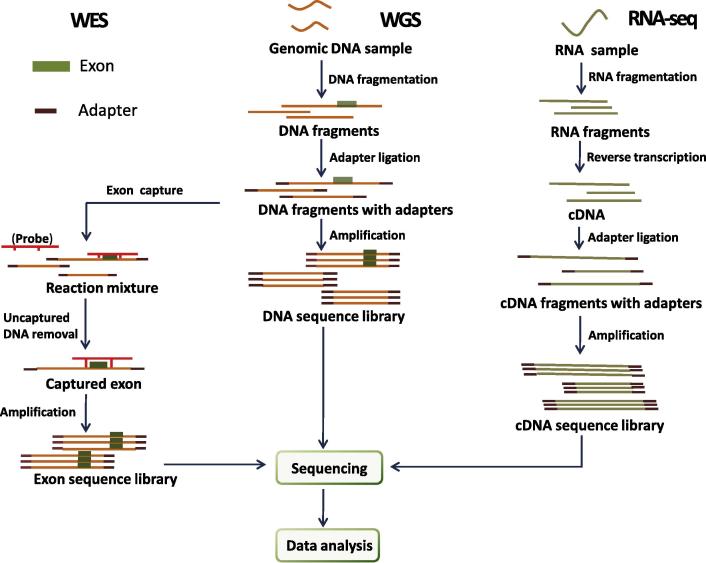
**Flow chart of common next generation sequencing approaches** **WES:** Workflow for WES. Genomic DNA samples are broken up into short-length fragments. After adaptor ligation, exonic DNA fragments are captured with exon-specific probes and amplified by PCR to prepare the exon sequence library. **WGS:** Workflow for WGS. Genomic DNA samples are broken up into short-length fragments. After adaptor ligation, the DNA fragments with adaptor are amplified by PCR to prepare the DNA sequence library. **RNA-seq:** Workflow for RNA-seq. RNA samples are randomly broken up into short-length fragments. After reverse transcription, cDNA fragments are ligated to adaptors and amplified by PCR to prepare the cDNA sequence library. After the sequence library is set up for each approach, sequencing is then performed on designated sequencers followed by computational analysis. **WES**, whole-exome sequencing; **WGS**, whole-genome sequencing.

With the development of NGS technology, WGS, WES, and RNA-seq are widely used to study the genetic mutations and gene expression (Figure 1). The most popular NGS method is WES. WES facilitates the capture of all coding exons in the genome. The term “exome” refers to all the exons in the genome, which covers approximately 1% of the genome in human. Nonetheless, approximately 85% of disease-related mutations are found in the exome [15,16]. WES is an efficient way to detect novel disease-causing genes, such as *MALT1* and *ACT1* [17,18], mutations of which can cause immunodeficiency diseases. Compared to WES, WGS facilitates sequencing of the whole genomes including both the coding and non-coding regions. As a result, WGS facilitates the identification of gene fusions and exon duplications, as well as detection of other genetic defects that might be missing by WES [19]. After optimizing the sequencing procedure, Mardis et al. successfully sequenced the whole cancer genome of an acute myeloid leukemia patient with increased sequencing coverage, fewer runs, and reduced false positive as compared to a previous report [20]. Their study provides the opportunity for the use of WGS in the detection of other complicated diseases such as autoimmune diseases. However, there are disadvantages associated with WGS, including high cost and long time required for bioinformatic analysis [19]. In recent years, the decreased cost and newly-developed techniques for bioinformatic analysis have resulted in wide application of WGS for the analysis of systemic autoimmune diseases. Abnormal RNA expression is closely related to the development of many diseases. NGS-based RNA-seq is used to sequence the total RNA to detect the change of gene expression. Recent studies on small RNAs (sRNAs), especially microRNAs (miRNAs), have uncovered some causative links between sRNAs and complicated diseases. Although miRNAs cannot be translated, they regulate over half of all protein-coding transcripts and function in the pathogenesis of diseases. RNA-seq is also used to discover novel miRNAs due to its low background and high sensitivity [21,22]. The application of RNA-seq is limited due to the fragile nature of RNA. With careful preparation, this technique will be very powerful to analyze the entire transcriptome including differential splicing and allelic expression [23,24]. Genetic variants related to autoimmune diseases identified or confirmed by NGS are summarized in Table 1, while the gut microbiotas related to autoimmune diseases identified by NGS are listed in Table 2.

## Applying NGS to SLE

SLE is a complex and heterogeneous disease involving both genetic and environmental factors. Multiple susceptibility genes have been identified to be associated with SLE. However, the correlation of some genes with SLE needs to be validated. NGS technology provides scientists with a good opportunity to evaluate the validity of previously-identified genes. In a study to evaluate the contribution of *IRF2* polymorphisms to SLE in an Asian population, Kawasaki et al. re-sequenced the *IRF2* genes using the 454 sequencing platform. The WGS results demonstrated that the rs66801661A gene variant could independently contribute to SLE. Additionally, it was observed that both rs66801661 and rs6233999 variants were correlated with transcriptional activation of *IRF2* [25]. This study rectified the previous interpretation that the *IRF2* correlation with SLE depended on other factors, demonstrating the power of NGS in validation of previously-identified genes. In addition, traditional sequencing did not allow for sufficient analytical accuracy for small sample size, while NGS can facilitate scientists in profiling transcription of disease-related genes in small sample populations. For instance, *IRF5* gene has been confirmed as a genetic risk factor for SLE by RNA-seq [26]. Analysis of the full-length *IRF5* transcription unit by RNA-seq from six patients and three healthy donors confirmed that the *IRF5* transcription profile differed in SLE patients compared to the controls. The abnormal IRF5 transcriptional signature was determined by *IRF5-*SLE risk haplotype [27]. A Solexa deep sequencing on 40 peripheral blood mononuclear cell (PBMC) samples from SLE patients and healthy controls revealed 61 novel miRNAs, such as hsa-miR-5683, that displayed different expression levels in SLE patients when compared to the controls. Genes targeted by these miRNAs function in cell metabolism and are likely related to the risk of SLE [28]. RNA-seq is an ideal tool to attain more in-depth information when using only small amounts of material.

Although human genetic factors have attracted most attention, there is emerging evidence supporting that the bacterial flora contribute to the pathogenesis of SLE. Culture-independent, high-throughput NGS technologies enable us to investigate the function of the gut microbiome in diseases. Sequencing of the 16S rRNA gene variable region using NGS technology has been used to distinguish and classify bacteria. For instance, 16S rRNA gene sequencing in combination with NGS was used to assess fecal microbial profiles of SLE patients and matched controls, revealing some significantly-decreased Firmicutes families and a lower Firmicutes/Bacteroidetes ratio in SLE individuals compared to controls [29]. This is the first report to show that dysfunction of the immune system in SLE patients may influence the gut microbiome community. Another group employed the Illumina platform and showed the presence of increased Clostridiaceae and Lachnospiraceae populations in MRL/lpr mice during disease progression [29]. The gender-specific alteration of disease status due to the presence of certain bacterial phylotypes (such as Lachnospiraceae) in the gut microbiota may be associated with the degree of severity of lupus symptoms in female MRL/lpr mice [30]. Most recently, a NGS-based study was performed to detect the impact of drinking water pH on the gut microbiome in association with disease development in (SWR × NZB)-F1 (SNF1) spontaneous lupus mice. Sequencing of fecal samples of diseased mice showed that individual mice that drank acidic pH water (AW) possess higher levels of *Lactobacillus reuteri* and *Turicibacter* spp. than those ingested neutral pH water (NW). The relative proportions of some bacterial species such as *Ruminococcus gnavus*, *Trichodesmium hildebrandtii*, *Hydrocarboniphaga daqingensis*, and *Polaribacter butkevichii* were higher in AW recipients than in NW recipients even in the pre-disease stage [31]. Although traditional sequencing technologies such as Sanger sequencing can help facilitate studies of the gut microbiome, they are time-consuming and unable to detect low frequency bacteria. With the high efficiency, high sensitivity, and high throughput properties associated with NGS, scientists are now capable of in-depth characterizing the components of the intestinal microbiome. Previous culture-based studies and studies detailed in this review have shown that gut bacterial populations might be correlated to the pathogenesis of SLE. Future gut microbiome studies associated with SLE should focus on the correlation of the relative change in proportion of bacteria with the mucosal immune response.

## NGS application and RA

RA is a chronic complex genetic autoimmune disorder characterized by synovial inflammation and erosion of bone and cartilage, which affects around 1% of the world’s population [32]. Klarenbeek and his colleagues conducted a series of experiments to characterize the T-cell receptor (TCR) and B-cell receptor (BCR) repertoire using RNA-seq. Through sequencing of TCR from synovial samples of patients with recent onset or established RA, more than 10,000 TCRs were attained per sample. This study demonstrated that the T cell repertoire was dominated by highly-expanded clones in early RA synovium. Such clonal dominance was more obvious than that observed in samples from patients with established RA [33]. Using the same protocol, the BCR repertoire in the synovium and blood of patients with early and active stage RA was sequenced to identify autoreactive B-cell and plasma-cell clones related to the disease status. It was shown that the dominant synovial clones with longer complementary-determining region 3 (*CDR3*) and immunoglobulin heavy chain (*IgH*) gene segment V4–34 enrichment were associated with RA severity [34]. As demonstrated by these two aforementioned studies, high sensitivity associated with NGS has permitted sequencing low blood or synovium cell quantities, which has been previously proven difficult using traditional protocols. Additionally, NGS allows screening clone activity quantitatively, which was previously difficult using Sanger sequencing.

NGS is also very powerful in assessing variants that occur at low frequencies. A group of rare non-synonymous variants was found in the *IL2RA* and *IL2RB* genes in RA patients in a European project. WES studies identified a missense variant (rs699738) and a non-coding variant (rs624988), which are believed to contribute to the risk of RA development [35]. Recently, a WES was performed in a 4-generation consanguineous pedigree study. A novel single missense mutation (p.G755R) within the *PLB1* gene locus and two independent non-coding variants (rs116018341 and rs116541814) close to *PLB1* gene were identified to be associated with the risk of RA development, indicating that *PLB1* is a susceptibility gene for RA [36]. Although GWAS allows for the identification of susceptibility variants, the associated low sensitivity limits its application. NGS technology, with high sensitivity and high-throughput properties, results in the provision of more convincing data, which cannot be obtained using GWAS.

Scher et al. sequenced V1 and V2 region of 16S rDNA on the Roche 454 platform by shotgun sequencing. They found a significant increase in *Prevotella copri* at the level of family abundances in new-onset untreated RA (NORA) patients. Such increase in *P. copri* is strongly correlated with disease progression. Interestingly, with increased amount of *Prevotella*, there is a concomitant reduction in abundance of *Bacteroides* in RA patients [37]. Recently, in collagen-induced arthritis mice, gut microbiota was found to promote the differentiation of IL-10, producing regulatory B cells in the spleen and mesenteric lymph nodes [38]. Both of these studies sequenced the different variable regions of 16S rDNA using fecal material on high-throughput NGS platforms. Most recently, a clinical research detected dysbiosis in RA patients’ oral and gut microbiomes in comparison to healthy controls. The dysbiosis was remitted to a certain extent after treatment with disease-modifying antirheumatic drugs (DMARDs). Fecal, dental plaque, and salivary samples were subjected to paired-end metagenomic sequencing on the Illumina platform. The relative abundance of *Haemophilus* spp. was found to decrease in RA individuals, whereas abundance of *Lactobacillus salivarius* showed a significant increase in active RA patients [39]. The appraisable outcome of this study provides the potential application of microbiome composition in clinical prognosis and diagnosis of RA. To sum up, NGS provides a powerful tool to examine and characterize microbial profiles, which are vital clues in uncovering the pathogenesis of RA. Additional studies and analysis are necessary to understand the interplay between microbial communities and the host in RA.

## The application of NGS in the study of MS

MS is a chronic autoimmune disease of the central nervous system (CNS) involving dysfunction of the blood–brain barrier in association with demyelination and axonal damage [40]. A recent study reported WGS of the Dark Agouti (DA) rat and control strains on the SOLiD platform. A single nucleotide variation was identified in a regulatory region of the *IL21R* gene, which is associated with MS [41]. Using the Illumina platform, Ramagopalan et al. sequenced 43 MS patients by WES, and identified a single rare variant (rs11820400) in the *CYP27B1* gene, which can cause loss of gene function, resulting in vitamin D deficiency-induced MS. The *CYP27B1* gene was also identified as a MS causative gene [42]. Another study focused on a family with high frequency of MS, which contains 15 individuals with MS in four consecutive generations. Among them, four family members were enrolled in the study for WES using the Illumina genome analyzer platform. Over 20,000 shared variants were identified in these family members, along with a rare mutation of rs55762744 in the *TYK2* gene encoding tyrosine kinase 2. Interestingly, rs34536433, which was previously reported to be associated with MS, was not found as a risk-factor for MS in this study [43]. In summary, WES provides a fast and cost-effective platform to detect rare variants in limited sample size, which cannot be achieved by the traditional Sanger sequencing technologies.

Epstein–Barr virus (EBV) has been reported to play an important role in the pathogenesis of MS [44]. Sequencing of the TCR repertoire by RNA-seq in the cerebrospinal fluid and blood of MS patients confirmed that the EBV-reactive CD8^+^ T cells are intrathecally enriched in MS patients only [45]. A recent study based on amplicon deep sequencing demonstrated that among the EBV alleles, the 1.2 allele is dominant in MS patients compared to healthy control. Several novel variants at nucleotides 402, 708, 733, and 800 were detected in the 1.2 allele. These variants were associated with the risk of MS development. In this study, NGS not only confirmed previous Sanger sequencing results, but also produced data that solved previous controversy regarding the association of Epstein–Barr nuclear antigen 2 (EBNA-2) with MS [46]. The advantage of NGS in the detection of cells that are present low in the peripheral blood provides us with a more sensitive method for sequencing low-copy mutations.

Many studies have also shown that gut microbiota plays an important role in the pathogenesis of MS. *Polysaccharide* A (PSA) produced by intestinal *Bacteroides fragilis* has been discovered to protect against the disease symptoms in the experimental autoimmune encephalomyelitis (EAE) model [47]. *Clostridium perfringens* type B was found to enhance nascent MS lesion formation [48]. Currently, there are no reports using NGS-based technology to study the distribution and function of the microbiome in relation to the pathogenesis of MS. Affymetrix PhyloChip arrays have been used to study intestinal bacteria associated with MS. Nonetheless, hundreds of unknown gut colonizing bacteria are not identified, since PhyloChip can only detect known bacteria [49]. Conversely, NGS allows sequencing of most gut microbial species. Therefore, this method provides a powerful tool to identify new strains of gut bacteria related to the pathogenesis of MS in future.

## NGS application and other systemic autoimmune diseases

In addition to SLE, MS, and RA, NGS has also been applied in the studies related to other systemic autoimmune diseases, including AS and SS. AS is an autoimmune disorder with chronic inflammation affecting both the spine and joints. Previous studies showed that the dysbiosis of the gut microbiome in AS is correlated with the presence of human leukocyte antigen B27 (HLA-B27), which is an AS disease-risk factor [50]. Using a HLA-B27 transgenic (Tg) Lewis rat model, 16S rRNA amplicon sequencing was used to detect HLA-B27 associated bacteria. This study demonstrated a significant difference in cecal microbiome between HLA-B27 Tg and wild type control rats. The increased population of *Paraprevotella* and *Bacteroides vulgatus* in Tg rats further confirmed that the HLA-B27 is associated with an altered gut microbiome [51]. Most recently, a clinical study sequenced the terminal ileum microbiome in nine patients with new-onset AS and revealed increased levels of Lachnospiraceae, Ruminococcaceae, Rikenellaceae, Porphyromonadaceae, and Bacteroidaceae, along with decreased number of Veillonellaceae and Prevotellaceae. The altered microbial composition is likely associated with risk of AS [52]. Both studies employed 16S rRNA gene sequencing using the Illumina MiSeq sequencer, demonstrating the advantages of NGS technology in aiding to identify more disease-related gut bacterial strains and the function of bacteria on the pathogenesis of AS.

SS is another complex autoimmune disease that causes exocrine gland deficiency along with symptoms of dryness with unknown etiology. Abnormal miRNA expression patterns could serve as the potential biomarker for SS [53]. Deep sequencing of small RNAs from SS patients and healthy controls using the SOLiD 4 platform resulted in the discovery of six novel miRNAs including hsa-miR-4524b-3p, hsa-miR-4524b-5p, hsa-miR-5571-3p, hsa-miR-5571-5p, hsa-miR-5100, and hsa-miR-5572. Among them, hsa-miR-5100 was found to be significantly correlated with SS [54]. With the application of NGS-based sequencing, more miRNAs will be identified, which would potentially facilitate the diagnosis of SS in future.

## Conclusions and perspectives

The rapid development of NGS has contributed significantly to both basic and clinical studies. The discovery of causative gene loci has given us the chance to unravel the factors involved in pathogenesis of diseases. Many disease-associated genes, which were previously missed due to the limitation of sequencing technology, have been identified by NGS. Gene identification is an important step in the discovery of the pathogenesis of the complicated autoimmune diseases. As mentioned above, NGS has facilitated validation of the association of previously-identified disease-related gene loci with autoimmune diseases. In addition, NGS enables researchers to combine previously-identified gene linkage with new sequencing data, allowing the discovery of previously-undetected linkage signals or pathways. NGS has also resulted in the correction of some previously-controversial linkages of susceptibility genes with diseases. Although WES is currently the main method for detecting rare variants in systemic autoimmune diseases, the dropping cost of WGS will allow for further identification of non-coding variants related to the risk of diseases. In addition to basic studies, application of NGS will also significantly improve the genetic diagnosis of autoimmune diseases, by providing clinicians with more accurate genetic information of the patients and assisting them in the formulation of a much more personalized and precise treatment regimen.

Increasing evidence has demonstrated the role of the gut microbiota in systemic autoimmunity [52]. Taking into account the complexity of systemic autoimmune diseases, in addition to identify the susceptibility genes, investigating the gut microbiome will help advance our understanding of disease development as well. Extensive studies need to be performed to identify the specific bacterial species that are related to host genetic mutations in systemic autoimmunity. Environment–gene interaction may affect genetic alleles, indicating that the gut microbiome is likely to play an important role in the genetic mutation of genes related to systemic autoimmune diseases [55]. Traditional technologies primarily rely on culture-dependent methods, which require different media types to culture target bacteria from fecal samples that contain a large number of bacterial species. Nonetheless, such technologies can only identify approximately 10%–15% of bacterial species, whereas the majority of bacterial species in the gut microbiota that function in gut microenvironment maintenance cannot be detected due to the difficulties in culturing such bacteria [56]. Due to the time-consuming and complexity nature of such methods, the culture-dependent methods have limited application in microbiome research. On the other hand, culture-independent approaches, which overcome the aforementioned limitations, are widely applied in today’s microbiome studies. For instance, 16S rDNA sequencing based-NGS can be applied to most microbiome diversity studies. Moreover, data generated using these methods can be used to gain further understanding of the disease development. Terminal restriction fragment length polymorphism (T-RFLP) is a method used to detect the length variation of the terminal restriction fragments of conserved genes such as 16S rRNA gene [57] and has shown some potential to generate more accurate data for 16S rDNA. T-RFLP is also sensitive, which allows for the detection of small amounts of intestinal bacteria [58]. Future microbiome studies are likely to combine NGS technology with T-RFLP to allow for more sensitive and reliable analyses. This will help scientists unravel the pathogenicity associated with the gut microbiota in relation to the disease development and ultimately explain the interplay between the microbiome and host genetic factors.

Epigenetic markers are closely related to the intestinal homeostasis and can be analyzed by NGS technologies including RNA-seq, ChIP sequencing, and DNA methylation sequencing. Due to limitations of traditional sequencing technologies, there are barely any reports using integrative analysis to study the inter-relationship between genomic mutations, cytokine expression, and the gut microbiome in autoimmune diseases. With the development of NGS technology, a comprehensive analysis that will aid in the understanding of the pathogenesis of systemic autoimmune diseases involving genetic factors, microbiome community, and cytokine network in autoimmunity is now possible. The genetic pedigrees in systemic autoimmune diseases will provide us with ideal candidates to study the correlation between genetic mutations and the alteration of the gut microbiome using NGS.

Although NGS technology has been widely used in autoimmune disease studies, there are some limitations. NGS technology is primarily based on short reads and produces large amounts of sequencing data. This is a big challenge for data analyses. It is also inevitable that NGS will bring in some errors, including the lack of detection on low frequency mutations due to the shorter lengths and repeat base sequences. Roche has developed a new GS FLX^+^ system, which can reach up to 1000 bp read length, overcoming short read limitations [59]. Bioinformatic analysis is also a time-consuming step, which can limit the application of NGS. In addition, biological databases are expanding due to the increasing number of genetic variation studies and microbiome studies. This brings the challenge of interpreting data appropriately. With the development of computational technology, more powerful software will reduce the cost and time required for the data analyses, and help interpret the sequencing data precisely. It is likely that NGS technology will become the main platform in the future studies of autoimmune diseases by overcoming problems associated with cost, library preparation, reading error, and extensive bioinformatic analysis.

## Competing interests

The authors have declared no competing interests.

## Figures and Tables

**Table 1:** Variants associated with systemic autoimmune diseases identified or validated by NGS

**Method**	**Disease**	**Gene/gene locus**	**Variant**	**Ref.**
WGS	SLE	*IRF2*	rs66801661, rs62339994	[25]
WGS	MS	*IL21R*	Eae29	[41]
WES	RA	*CD2*	rs699738, rs624988	[35]
	*IL2RA*	Unidentified	[35]
	*IL2RB*	Unidentified	[35]
	*PLB1*	rs116018341, rs11651814, P.G755R	[36]

WES	MS	*CYP27B1*	rs118204009	[42]
	*TYK2*	rs55762744	[43]

*Note:* WGS, whole-genome sequencing; WES, whole-exome sequencing; SLE, systemic lupus erythematosus; MS, multiple sclerosis; RA, rheumatoid arthritis; IRF-2, interferon regulatory factor 2; IL21R, interleukin 21 receptor; CD2, cluster of differentiation 2; IL2RA, interleukin 2 receptor, alpha; IL2RB, interleukin 2 receptor, beta; PLB1, phospholipase B1; CYP27B1, cytochrome P450, family 27, subfamily B, polypeptide 1; TYK2, tyrosine kinase 2.

**Table 2:** Alteration in gut microbiota related to autoimmune diseases as identified by NGS

**Disease**	**Fecal sample**	**Alteration in microbiota**	**Ref.**
SLE	SLE patients	Firmicutes↓ Firmicutes/Bacteroidetes↓	[29]
	MRL/lpr mice	Clostridiaceae↑ Lachnospiraceae↑	[30]
	SNF1 lupus mice	*Lactobacillus reuteri*↑ *Turicibacter* spp.↑	[31]

RA	RA patients	*Prevotella*↑ *Bacteroides*↓	[37]
	RA patients	*Haemophilus* spp.↓ *Lactobacillus salivarius*↑	[39]

AS	Transgenic Lewis rats	*Paraprevotella*↑ *Bacteroides vulgatus*↑	[51]
	AS patients	Lachnospiraceae↑ Ruminococcaceae↑ Rikenellaceae↑ Porphyromonadaceae↑ Bacteroidaceae↑ Veillonellaceae↓ Prevotellaceae↓	[52]

*Note:* SLE, systemic lupus erythematosus; RA, rheumatoid arthritis; AS, ankylosing spondylitis. The increased and reduced abundance of the specified bacteria is indicated with ↑ and ↓, respectively.
